# Boron bridging of rhamnogalacturonan-II in *Rosa* and arabidopsis cell cultures occurs mainly in the endo-membrane system and continues at a reduced rate after secretion

**DOI:** 10.1093/aob/mcac119

**Published:** 2022-09-16

**Authors:** Rifat Ara Begum, Stephen C Fry

**Affiliations:** The Edinburgh Cell Wall Group, Institute of Molecular Plant Sciences, The University of Edinburgh, Daniel Rutherford Building, The King’s Buildings, Max Born Crescent, Edinburgh EH9 3BF, UK; Department of Biochemistry and Molecular Biology, Faculty of Biological Sciences, University of Dhaka, Curzon Hall, Dhaka – 1000, Bangladesh; The Edinburgh Cell Wall Group, Institute of Molecular Plant Sciences, The University of Edinburgh, Daniel Rutherford Building, The King’s Buildings, Max Born Crescent, Edinburgh EH9 3BF, UK

**Keywords:** Boron bridges, borate diesters, rhamnogalacturonan-II, pectin, cell-wall polysaccharides, radiolabelling, polyacrylamide gel electrophoresis, cell-suspension cultures, *Arabidopsis thaliana*, *Rosa* sp. (‘Paul’s Scarlet’)

## Abstract

**Background and aims:**

Rhamnogalacturonan-II (RG-II) is a domain of primary cell-wall pectin. Pairs of RG-II domains are covalently cross-linked via borate diester bridges, necessary for normal cell growth. Interpreting the precise mechanism and roles of boron bridging is difficult because there are conflicting hypotheses as to whether bridging occurs mainly within the Golgi system, concurrently with secretion or within the cell wall. We therefore explored the kinetics of RG-II bridging.

**Methods:**

Cell-suspension cultures of *Rosa* and arabidopsis were pulse-radiolabelled with [^14^C]glucose, then the boron bridging status of newly synthesized [^14^C]RG-II domains was tracked by polyacrylamide gel electrophoresis of endo-polygalacturonase digests.

**Key results:**

Optimal culture ages for ^14^C-labelling were ~5 and ~1 d in *Rosa* and arabidopsis respectively. *De-novo* [^14^C]polysaccharide production occurred for the first ~90 min; thereafter the radiolabelled molecules were tracked as they ‘aged’ in the wall. Monomeric and (boron-bridged) dimeric [^14^C]RG-II domains appeared simultaneously, both being detectable within 4 min of [^14^C]glucose feeding, i.e. well before the secretion of newly synthesized [^14^C]polysaccharides into the apoplast at ~15–20 min. The [^14^C]dimer : [^14^C]monomer ratio of RG-II remained approximately constant from 4 to 120 min, indicating that boron bridging was occurring within the Golgi system during polysaccharide biosynthesis. However, [^14^C]dimers increased slightly over the following 15 h, indicating that limited boron bridging was continuing after secretion.

**Conclusions:**

The results show where in the cell (and thus when in the ‘career’ of an RG-II domain) boron bridging occurs, helping to define the possible biological roles of RG-II dimerization and the probable localization of boron-donating glycoproteins or glycolipids.

## INTRODUCTION

Rhamnogalacturonan-II (RG-II) is a highly complex polysaccharide domain which, together with RG-I, homogalacturonan and sometimes xylogalacturonan, constitutes the pectin of the primary cell-wall matrix. In its monomeric form RG-II typically comprises 29–33 monosaccharide residues: up to 12 d-galacturonic acid (GalA), up to five l-rhamnose, up to four l-arabinofuranose, two d-apiose, and one each of l-arabinopyranose, l-fucose, 2-*O*-methyl-l-fucose, 2-*O*-methyl-d-xylose, d-aceric acid, d-glucuronic acid, d-galactose, l-galactose, a deoxyheptulosaric and a ketodeoxyoctulosonic acid (Pabst *et al.*, 2013; Ndeh *et al.*, 2017). Its structure is highly, though not absolutely, conserved across diverse land-plants.

Boron is an essential element in plant nutrition (Wimmer *et al.*, 2019). Plants obtain their boron in the form of un-ionized boric acid, which is soluble in the soil-water. Boron deficiency or excess can affect the mechanical properties of plants (Loomis and Durst, 1992; Blevins and Lukaszewski, 1998), and hence its relationship with the cell wall has been studied extensively (Hu and Brown, 1994; Hu *et al.*, 1996; Ishii and Ono, 1999). Boron’s best-established role in plants is as a covalent cross-link between RG-II domains, whereby the boron atom forms a borate diester with a specific apiose residue in each of two participating RG-II domains (Kobayashi *et al.*, 1996; O’Neill *et al.*, 1996, 2001; Ishii *et al.*, 1999, 2002). The *mur1* mutant of *Arabidopsis thaliana*, which produces RG-II with a truncated sidechain A, is unable to form a stably boron-bridged RG-II dimer (O’Neill *et al.*, 2001), though its phenotype can be returned to resemble that of the wild-type by an elevated boron supply (Panter *et al.*, 2019).

One role of RG-II dimerization is to correctly adjust wall porosity (Fleischer *et al.*, 1999). However, the precise role of boron bridges in RG-II with regard to growth is difficult to define. On the one hand, by cross-linking neighbouring pectin chains, boron bridging might be involved in assembling the cell-wall’s architecture and perhaps in tightening the cell wall (Ishii *et al.*, 2001), thus decelerating cell expansion. On the other hand, the prevention of boron bridging (either by boron starvation or by genetic modification of the structure of RG-II) results in diminished cell growth (O’Neill *et al.*, 2004), pointing to boron bridges accelerating cell expansion. Whichever of these ideas prevails, boron is clearly essential for plant life, and its ability to cross-link RG-II domains is presumed to be its principal botanical role.

Potentially shedding light on the above conundrum, it is of interest to know when during its ‘career’ an individual RG-II domain is subject to boron bridging, and (a closely related question) where in the cell the dimerization occurs. Chormova *et al.* (2014) showed that the boron bridging of RG-II occurs during polysaccharide synthesis and/or secretion but not after the polysaccharide has arrived at its destination in the cell wall. This was demonstrated by use of polyacrylamide gel electrophoresis (PAGE) to separate monomeric from dimeric (=boron-bridged) RG-II. *Rosa* cells, cultured in the absence of boron, produced only monomeric RG-II; the re-addition of 3.3 µm boric acid triggered a very gradual appearance of the RG-II dimer over the following 24 h, but without detectable loss of existing monomers, suggesting that only newly synthesized RG-II domains are amenable to boron bridging. In agreement with this, *Rosa* cultures whose polysaccharide biosynthetic machinery had been compromised (by carbon starvation, respiratory inhibitors, freezing/thawing, etc.) lost the ability to generate RG-II dimers even when 3.3 µm boric acid was present in the medium. It was concluded that RG-II normally becomes boron-bridged during synthesis and/or secretion, but not post-secretion. Supporting this conclusion, exogenous radiolabelled RG-II, when added to *Rosa* cultures, was neither dimerized in the culture medium nor cross-linked to existing wall-bound RG-II domains. [Judging by the large size of soluble extracellular polysaccharides that are released from plant cells into the culture medium (Kerr and Fry, 2003), we expect that a 5-kDa RG-II molecule would be able to permeate the cell wall and come into contact with the plasma membrane. The ‘exclusion limit’ of the cell-wall matrix has been estimated by different methods to be ~5 kDa (Carpita *et al.*, 1979) and ~50 kDa (Tepfer and Taylor, 1981).] Therefore, it was concluded that, in cultured *Rosa* cells, RG-II domains have a brief window of opportunity for boron-bridging intraprotoplasmically and/or during secretion, but that secretion into the apoplast is a point of no return beyond which additional boron-bridging does not readily occur (Chormova *et al.*, 2014).

In contrast to this conclusion, it had been reported that when boric acid was re-supplied to boron-starved *Chenopodium* cells (Fleischer *et al.*, 1999) or *Cucurbita* leaves (Ishii *et al.*, 2001), many of the existing RG-II domains rapidly became boron-bridged. These results suggest that bridging can occur in the cell wall long after the pectin molecule containing a particular RG-II domain has been deposited in the wall, in contrast to the findings of Chormova *et al.* (2014).

In an attempt to resolve this discrepancy, we have now investigated in more detail the kinetics of boron bridging of RG-II domains in two different cell-suspension cultures which had been adapted over many years to very different boric acid concentrations. We pulse-labelled *Rosa* and arabidopsis cells (grown with 3.3 and 100 µm boric acid respectively) with a trace amount of [^14^C]glucose, from which the radioactivity is quickly incorporated into newly synthesized polysaccharides. This enabled us to track a cohort of labelled RG-II domains of known ‘age’ (time elapsed since synthesis), using gel electrophoresis to follow their subsequent dimerization *in vivo*.

## MATERIALS AND METHODS

### Cell-suspension cultures

Cell-suspension cultures of ‘Paul’s Scarlet’ rose (*Rosa* sp.) and arabidopsis (*Arabidopsis thaliana*; var. erecta) were sourced and maintained as described by Popper and Fry (2008), except that both cultures were maintained with 2 % glycerol as carbon source (which expedites the uptake and utilization of trace amounts of added [^14^C]glucose). The *Rosa* and arabidopsis cultures were maintained in 250- and 500-mL flasks, and their media contained 3.3 and 100 µm boric acid respectively. The large difference in boron concentrations is largely an accident of history. *Rosa* cultures had long been acclimated to a low boric acid concentration. It is not known why or how *Rosa* cultures can grow in low- (and even zero-) boron media, nor why the originators of this culture in October 1957 chose 3.3 µm as the boric acid concentration (Nickell and Tulecke, 1959). Chormova *et al.* (2014) showed that arabidopsis, unlike *Rosa*, cannot survive in low-boron media. Ultimately, this difference between the two cultures helpfully serves to establish that our conclusions apply equally to different plant species grown under different conditions.

### ^14^C-labelling of mini-cultures

For *in-vivo* radiolabelling, cultures of the ages (time after subculture) specified in the Figure legends were sieved through muslin (removing the larger cell aggregates) and allowed to sediment for ~10 min in a 15-mL graduated centrifuge tube, then sufficient culture medium was removed to leave ~0.5 mL of settled cells per millilitre. After resuspension, 100-µL aliquots were pipetted into 6-mL round-bottomed tubes (plugged with cotton wool), and these mini-cultures were shaken gently for 4 h, allowing the cells to acclimate after the shock of pipetting. [6-^14^C]Glucose (50 kBq) was then added to each mini-culture, giving a glucose concentration of 0.25 mm, and incubation with gentle shaking was continued for 0–1020 min.

### ^14^C uptake

After the required incubation period, the cells were sedimented and the culture medium (supernatant) was sampled. Portions were quantified for total ‘remaining’ extracellular ^14^C by scintillation counting in a Beckman LS5000 CE scintillation counter (Fullerton, CA, USA), and additional portions were analysed quantitatively and qualitatively by paper chromatography.

### Ethanol-soluble fraction and AIR

The cells were suspended in 75 % ethanol and incubated with stirring at 20 °C for 4–6 h. After centrifugation, portions of the ethanolic supernatant were assayed for total ^14^C by scintillation counting, and additional portions were analysed by paper chromatography. The alcohol-insoluble residue (AIR), expected to comprise polymers including cell-wall polysaccharides, was further washed three times in 95 % ethanol, then twice in 100 % acetone, and dried. Each 100-µL miniculture produced ~1 mg of AIR.

### Endopolygalacturonase digestion of AIR

The AIR was de-esterified in 1.0 m Na_2_CO_3_ (pH ≈ 11.5) at 4 °C for 16 h, then adjusted to pH 4.5 with acetic acid, rinsed with water followed by acetone, and dried. The solid material was digested with endo-polygalacturonase [EPG; 5 U mL^–1^; from *Aspergillus aculeatus*; Megazyme, http://www.megazyme.com; pre-dialysed against pyridine/acetic acid/water (1 : 1 : 98)] at 20 °C for 16 h. All water used for these treatments had been freed of soluble boron compounds on Amberlite IRA743 (Sigma, https://www.sigmaaldrich.com/). Portions of the digest were analysed by thin-layer chromatography (TLC) and PAGE.

### Paper and thin-layer chromatography, and paper electrophoresis

Paper chromatography was conducted on Whatman No. 3 paper in butan-1-ol/acetic acid/water (12 : 3 : 5 by vol.) by the descending method for 24 h. Strips of the chromatogram were assayed for ^14^C by scintillation counting. Non-radioactive marker glucose was stained with aniline hydrogen-phthalate (Fry, 2000).

TLC was performed on aluminium-backed Merck silica-gel layers in butan-1-ol/acetic acid/water (2 : 1 : 1; single ascent; ~8 h). The chromatogram was autoradiographed and non-radioactive markers were stained with thymol/H_2_SO_4_ (Jork *et al.*, 1994).

High-voltage paper electrophoresis was conducted on Whatman No. 3 paper in pH 2 buffer at 3.5 kV for 60 min in a white-spirit-cooled tank (Weigel, 1963; Fry, 2020). Non-radioactive marker sugars were stained with aniline hydrogen-phthalate (modified from Partridge, 1949), and UDP-glucose was located under a 254-nm ultraviolet lamp.

### Polyacrylamide gel electrophoresis

As described by Chormova *et al.* (2014), EPG digests (corresponding to the products from a specified weight of AIR) were analysed by PAGE. Total rhamnogalacturonans (radioactive + non-radioactive) were stained with silver nitrate, and ^14^C-labelled bands were visualized (after the gel had been dried) by autoradiography. Radioactive zones were cut from the dried gel and assayed for ^14^C by scintillation counting.

### Detection of radioactivity

Aqueous and ethanolic solutions were assayed for ^14^C by scintillation counting in 10 volumes of OptiPhase HiSafe 3 (PerkinElmer, Inc.) aqueous-miscible scintillant.

Gel electrophoretograms and TLCs were dried, then exposed to Kodak BioMax MR-1 film in the dark for ~4 weeks. Dry strips cut from paper chromatograms and paper electrophoretograms, and spots (localized by autoradiography) excised from TLCs, were assayed for ^14^C by scintillation counting in 2 mL of an aqueous-immiscible scintillant (Gold Star; Meridian Biotechnologies Ltd). Radioactive spots excised from gel electrophoretograms were hydrolysed in 2 m trifluoroacetic acid at 100 °C for 1 h, then assayed for ^14^C by scintillation counting in 10 volumes of aqueous-miscible scintillant.

## RESULTS

### Pulse-radiolabelling to determine the optimal age of cultures for incorporation of ^14^C into RG-II

Before conducting detailed pulse-radiolabelling experiments to track the boron bridging of RG-II *in vivo*, we first tested small samples taken from *Rosa* and arabidopsis cultures of different ages (i.e. time after subculture) for their ability to utilize [^14^C]glucose during a 2-h pulse. The pulse-labelled mini-cultures were then tested for net ^14^C uptake and for ^14^C incorporation into low-*M*_r_ cellular metabolites, pectin and non-pectic polymers. The results thus gave a ‘2-h snapshot’ of the cells’ ability to take up [^14^C]glucose and metabolize it in cultures of different ages.

**Fig. 1.**
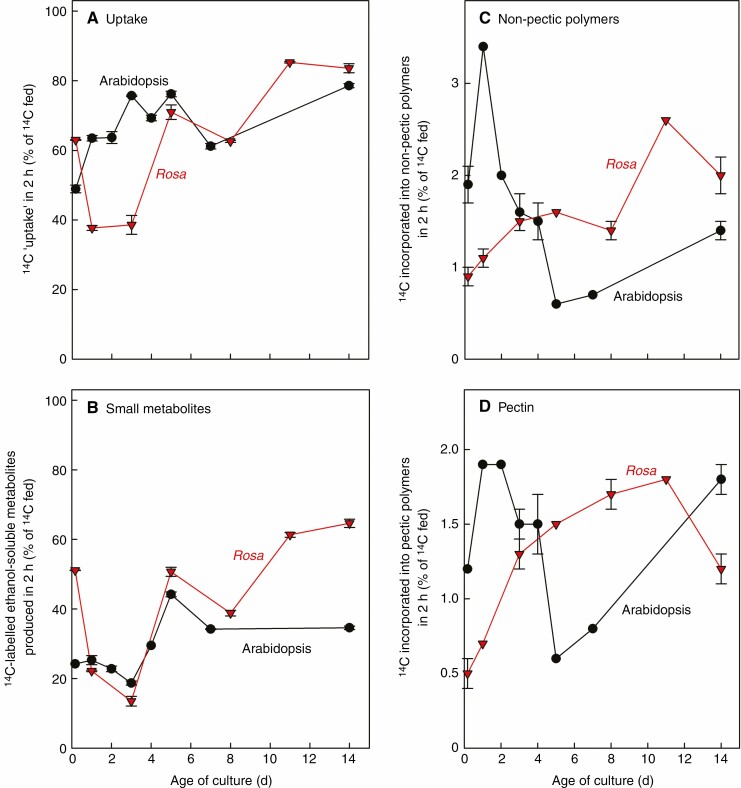
Uptake and metabolism of [^14^C]glucose by differently aged glycerol-grown cultures. Mini-cultures (100 µL in 6-mL tubes) of glycerol-grown rose and arabidopsis cells, sampled at 0 to 14 d after inoculation (always adjusted to 50 % settled cell volume and acclimated to the 6-mL vial for 4 h), were fed [6-^14^C]glucose (50 kBq; final concentration 0.25 mm) and incubated for a further 2 h. (A) Net uptake, estimated from ^14^C ‘remaining’ in the medium. (B) ^14^C-Incorporation into small intracellular metabolites. (C) ^14^C-Incorporation into non-pectic polymers (components not released from AIR by EPG). (D) ^14^C-Incorporation into pectin (components released from AIR by EPG). Error bars, where visible, show the range of two replicate mini-cultures.

Net ^14^C uptake was satisfactory at all ages (Fig. 1A). The cultures are routinely grown in medium containing glycerol as their carbon source, which promotes the rapid uptake and incorporation of any supplied traces of glucose.

^14^C-Accumulation into small (ethanol-soluble) intracellular metabolites after 2 h of incubation was also satisfactory at all ages (Fig. 1B), although these measurements fluctuate as they are the net result of [^14^C]glucose uptake vs. its onward metabolism (including loss of ^14^CO_2_ by respiration) and [^14^C]polymer synthesis. Thus, the cultures at all ages tested were metabolically active.

Accumulation of ^14^C in pectic domains (homogalacturonan and rhamnogalacturonans) was assayed as material solubilized from the AIR by EPG (Fig. 1D): it peaked in 1–2-d-old arabidopsis cultures (with an unexpected second peak at 14 d) and in 3–11-d rose cultures, approaching 2 % of the fed ^14^C. Labelling of non-pectic polymers (mainly cellulose, hemicelluloses, starch and proteins; ^14^C not solubilized from the AIR by EPG; Fig. 1C) broadly followed the same trends. The data show that [^14^C]pectin typically constituted 40–50 % of total [^14^C]polymers.

**Fig. 2.**
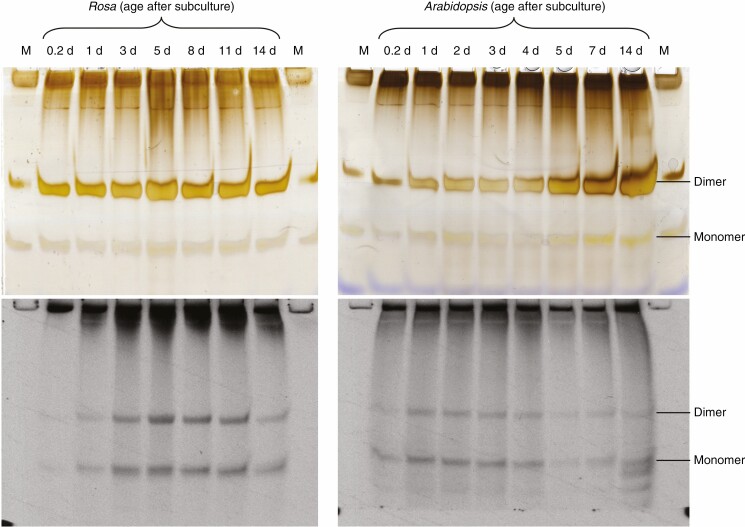
^14^C-Labelling of RG-II by differently aged cultures. Pulse-labelling with [^14^C]glucose for 2 h was as in Fig. 1. Portions of each EPG digest (see Fig. 1D) corresponding to the products obtained from 1.0 mg of AIR were analysed by gel electrophoresis: culture ages as shown above each lane. Left, *Rosa*; right, arabidopsis. Autoradiograms of dried gels (greyscale; 4 weeks of exposure) are aligned below the corresponding silver-stained gels (shown in colour). M: non-radioactive markers (0.8 µg monomeric plus 0.8 µg dimeric RG-II). A repeat of this experiment with mini-cultures taken from independent standard cultures is shown in Fig. S1.

The EPG-solubilized material (Fig. 1D) is expected to comprise RG-I, RG-II and oligogalacturonides (from homogalacturonan). RG-II labelling was assayed by gel electrophoresis followed by autoradiography (Fig. 2). RG-II was satisfactorily radiolabelled (during a 2-h pulse with [^14^C]glucose) in 3–11-d-old *Rosa* cultures, and in 1–3-d-old arabidopsis cultures – i.e. satisfactory for accurate quantification by the methods to be employed. Observing the intensity of radiolabelled RG-II bands on the autoradiograms, we selected 5-d-old *Rosa* culture and 1-d-old arabidopsis culture for future experiments. There was good agreement between duplicate mini-cultures (Fig. 2 and Supplementary Data, Fig. S1). Silver-staining showed that, in *Rosa*, little change in total RG-II (per mg AIR) or in the total monomer:dimer ratio occurred between 0.2- and 14-d-old cultures. In arabidopsis, there was a gradual increase in total RG-II between 0.2 and 14 d, most clearly seen in the dimer. From this experiment, it was concluded that 5-d-old *Rosa* and 1-d-old arabidopsis cultures are suitable for ^14^C-incorporation into RG-II.

### Tracking the boron bridging of RG-II in vivo by radiolabelling at optimal culture age

#### Uptake of ^14^C and net accumulation in pool of small metabolites

**Fig. 3.**
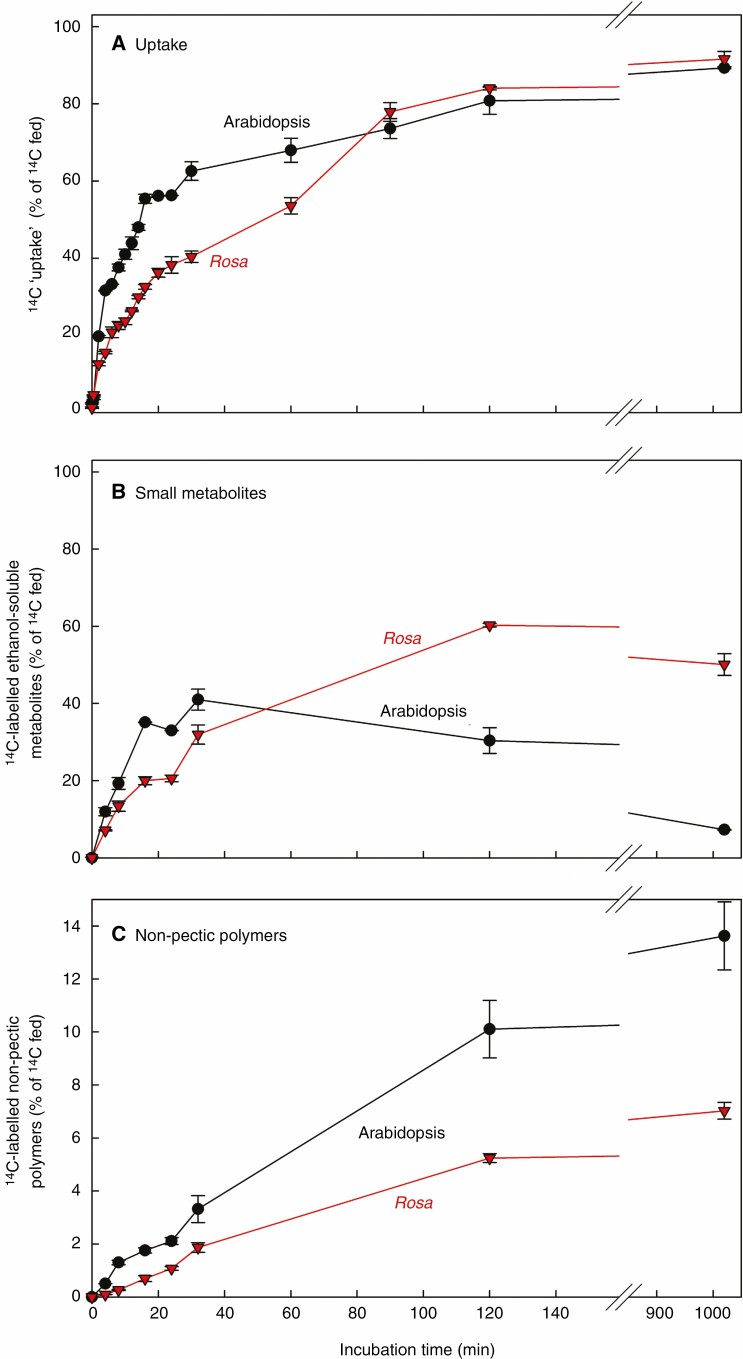
Net uptake and metabolism of [^14^C]glucose by *Rosa* and arabidopsis mini-cultures. Five-day *Rosa* and 1-d arabidopsis cultures were adjusted to 50 % (settled cell volume) SCV and dispensed as 100-µL ‘mini-cultures’ in 6-mL tubes. After 4 h acclimation in the new tubes, the mini-cultures were supplied with 50 kBq [6-^14^C]Glc (final concentration 250 µm). (A) Small portions of cell-free spent medium were collected at intervals and assayed for ^14^C. At other time-points, whole mini-cultures were sampled and assayed for (B) intracellular small (ethanol-soluble) metabolites and (C) non-pectic polymers (^14^C not released from de-esterified AIR by EPG). Data show the mean of two replicate mini-cultures ± range.

In experiments to study the kinetics of RG-II dimerization *in vivo*, we incubated cell-suspension cultures of optimum age (5-d *Rosa* and 1-d arabidopsis cultures) with a trace amount of [^14^C]glucose, and took samples at intervals. About 50 % of the radioactivity was ‘taken up’ from the medium within 50 min by *Rosa* cells and within 15 min by arabidopsis (Fig. 3A). Both species had removed ~90 % of the ^14^C by 1020 min. Uptake' (removal of [^14^C]glucose from the medium) is only slightly underestimated by the concurrent release of ^14^C-metabolites into the medium (Becker *et al.*, 1964; Aspinall *et al.*, 1969; Kerr and Fry, 2003; Fry, 2004): trace amounts of immobile radioactivity were observed at the origin of the paper chromatogram (i.e. −2 to +2 cm on the *x*-axis), especially at the last time-point, representing soluble extracellular [^14^C]polymers released from the cells (Supplementary Data, Fig. S2).

Large proportions (40–60 %) of the total ^14^C entered the pool of (ethanol-soluble) small metabolites [Fig. 3B; which would include organic acids, sugar-phosphates, etc.; some being subsequently lost as ^14^CO_2_ by respiration (Sharples and Fry, 2007)], and smaller proportions (4–10 %) were incorporated into non-pectic polymers (components of AIR not solubilized by EPG; Fig. 3C).

#### Kinetics of radiolabelling of homogalacturonan

**Fig. 4.**
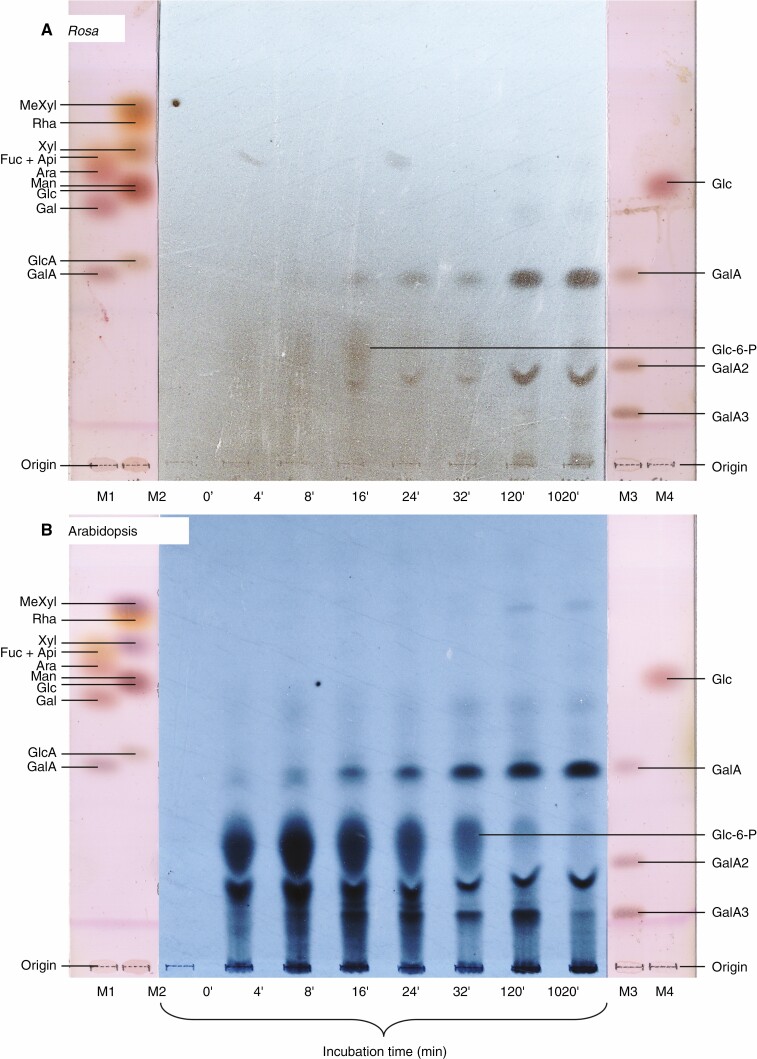
^14^C-Pectic fragments released by endo-polygalacturonase digestion. A sample of each EPG digest (corresponding to the products released from 0.125 mg of AIR; dried and re-dissolved in ethanol) was analysed by TLC in butan-1-ol/acetic acid/water (2 : 1 : 1; one ascent). (A) *Rosa*; (B) arabidopsis. Autoradiograms are shown alongside thymol-stained markers (M1–M4).

To specifically examine the production of [^14^C]pectic material, we digested the total polymers (AIR; after de-esterification) with EPG, which releases water-soluble fragments of pectin. The products are expected to include ethanol-insoluble rhamnogalacturonans plus (homogalacturonan-derived) ethanol-soluble oligogalacturonides. TLC of the latter revealed not only the expected [^14^C]GalA_1–3_, but also ^14^C-labelled sugar phosphates, especially at the early time-points in arabidopsis (Fig. 4). These sugar phosphates had evidently not been completely extracted by the ethanol used during the initial preparation of AIR, but did subsequently dissolve in the aqueous EPG solution and thereafter re-dissolved in ethanol. The major spot, which ran on TLC slightly faster than GalA_2_, was shown by paper electrophoresis at pH 2 to be [^14^C]glucose 6-phosphate (Glc-6-P; Supplementary Data, Fig. S3), the major sugar phosphate in cultured plant cells (Sharples and Fry, 2007) and whose phosphate group confers a strong negative charge, even at pH 2 (Fry, 2020). GalA_1–3_ are much slower-migrating on electrophoresis at pH 2 owing to their higher *p*K_a_ values (Al Hinai *et al.*, 2021). [Glc-6-P is the plant cell’s principal sugar phosphate quantitatively, and the major ^14^C peak detected (Fig. S3) co-migrated exactly with Glc-6-P on high-voltage electrophoresis (Fig. S3).] This methodology gives partial or complete resolution of Glc-6-P from most other relevant phosphates including AMP, ADP, ATP, NAD, NADP, dihydroxyacetone phosphate, ribulose 5-P, ribose 5-P, Glc-1-P, fructose 6-P, sedoheptulose 7-P and all sugar bisphosphates. Gluconate 6-P is the only important phosphate not distinguished from Glc-6-P under these electrophoretic conditions (see fig. 3a of Fry, 2020).]

The Glc-6-P spot very rapidly became radiolabelled (peaking at ~8 min; Fig. 4). It did not plateau but underwent turnover, as expected for an intermediary metabolite. This observation confirms that the cultures were effectively being pulse-labelled.

**Fig. 5.**
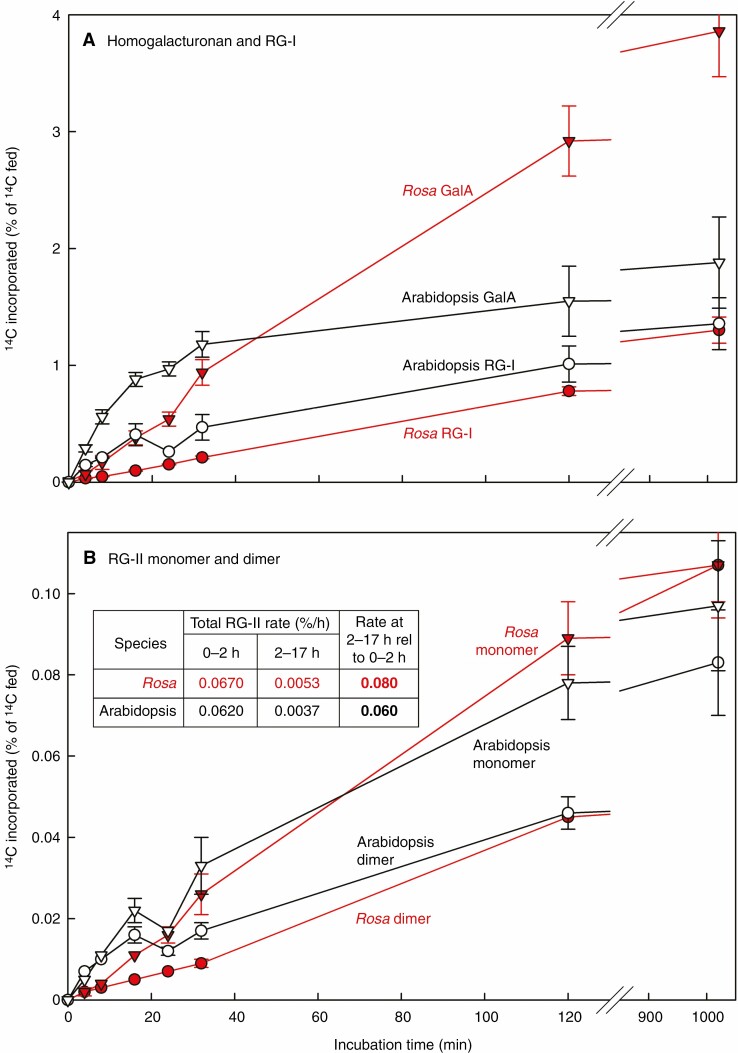
Quantification of ^14^C-labelling of pectic domains including monomeric and dimeric RG-II. Radiolabelling of (A) EPG-releasable GalA (quantified from TLC) and RG-I (from gel electrophoresis); (B) RG-II monomer and dimer (quantified from gel electrophoresis). After polyacrylamide gel electrophoresis (Fig. 6), the bands were cut out, acid-hydrolysed and assayed for ^14^C by scintillation-counting. Error bars show the range of the data; *n* = 2.

The presence of radiolabelled sugar phosphates in the EPG digest made it inappropriate to estimate total pectin by measuring the AIR material that became ethanol-soluble after EPG treatment. Instead, we assayed purely the EPG-released [^14^C]GalA_1_ monosaccharide, which after TLC was not contaminated by sugar phosphates (Fig. 4). All the EPG digests are expected to give GalA, GalA_2_ and GalA_3_ in the same ratio because these three products arise only from homogalacturonan, which we had de-esterified to make all samples fully EPG-digestible. Thus, the quantity of [^14^C]GalA_1_ is proportional to total oligogalacturonides, and therefore to homogalacturonan (Fig. 5A).

On this basis, in the early stages of the time-course (0–30 min in arabidopsis and 0–120 min in *Rosa*), both cultures were actively producing [^14^C]homogalacturonan, reaching ~1.5–3 % of the total supplied ^14^C. During the following 900 min any further increase was at a much lower rate (Fig. 5A). Such a rise to a plateau of radiolabelling is characteristic of a stable end-product, as expected for a cell-wall polysaccharide domain, synthesized during a labelling experiment.

#### Kinetics of radiolabelling of RG-I

**Fig. 6.**
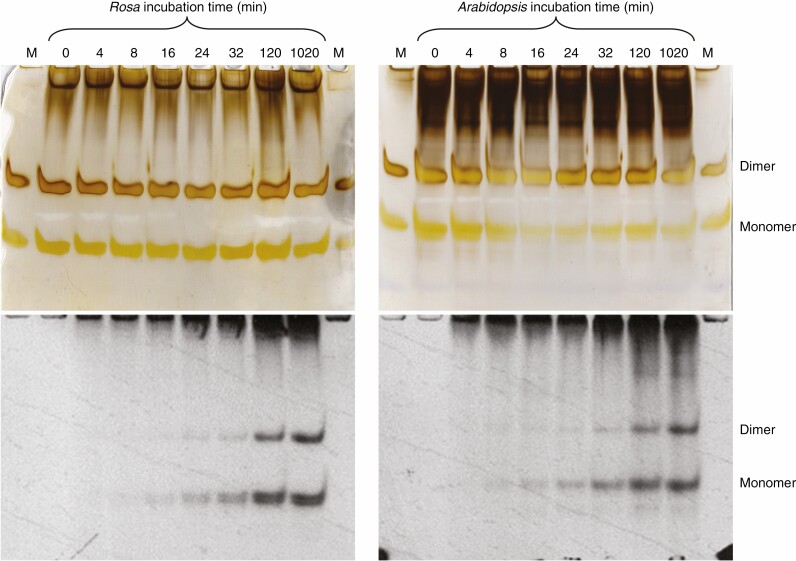
Time-course of ^14^C-labelling of monomeric and dimeric RG-II domains. Radiolabelling of mini-cultures with [^14^C]glucose for 0–1020 min was as in Fig. 3. Portions of each EPG digest (corresponding to the products obtained from 0.375 mg of AIR) were analysed by PAGE. Left, *Rosa*; right, arabidopsis. Autoradiograms (4 week of exposure) of the dried gels (shown in greyscale) are aligned below identical but silver-stained gels (shown in colour). M, non-radioactive markers (0.8 µg monomeric plus 0.8 µg dimeric RG-II). A repeat of this experiment with mini-cultures taken from independent standard cultures is shown in Fig. S4.

The EPG digests were further analysed by gel electrophoresis, revealing pulse-labelled [^14^C]RG-I and RG-II (by autoradiography) and total RG-II (by silver staining) (Fig. 6). [^14^C]RG-I labelling followed a trend similar to that of homogalacturonan (Fig. 5A), reaching ~0.7–1.0 % of the total fed ^14^C within 120 min, followed by an approximation to a plateau. This is the kinetics expected of a metabolic end-product.

#### Kinetics of radiolabelling of RG-II

On inspection of the same silver-stained gels, total RG-II (radioactive + non-radioactive) showed no consistent time-dependent changes. Both monomeric and dimeric RG-II were detectable (Fig. 6). Judged by silver staining, the dimer : monomer ratio was roughly 1 : 1 in *Rosa* (the same as in the marker mixture ‘M’; note that silver staining overestimates the dimer relative to monomer) and >1 : 1 in arabidopsis. The difference in ratio probably reflects the difference in boric acid concentration between the two culture media (3.3 and 100 µm for rose and arabidopsis, respectively).

^14^C-labelling of RG-II was detectable by autoradiography in both cultures by 4 min. Thus, the pathway from exogenous [^14^C]glucose to [^14^C]RG-II required <4 min. The labelling trends are visible qualitatively in Fig. 6. For quantification, the bands were assayed for ^14^C by scintillation-counting (Fig. 5B). The average rate for monomeric plus dimeric RG-II radiolabelling during this interval was ~0.06 % (of the supplied ^14^C) h^–1^ (Fig. 5B), mainly representing *de-novo* [^14^C]RG-II biosynthesis. Between 120 and 1020 min, during which period there was little remaining extracellular [^14^C]glucose, essentially no [^14^C]glucose 6-phosphate, and little further [^14^C]homogalacturonan and [^14^C]RG-I synthesis, the average rate of [^14^C]RG-II accumulation was reduced to about 0.013 % h^−1^, agreeing with the plateau seen in Fig. 3C.

Between 4 and 120 min, the radiolabelling of RG-II dimers paralleled that of the monomers (Figs 5B and 6). Both were detectable by 4 min after the feeding of [^14^C]glucose, showing that there was a negligible lag period between the synthesis of an RG-II domain and its cross-linking via a boron bridge.

However, between 120 and 1020 min (i.e. in the virtual absence of extracellular [^14^C]glucose and during a period when essentially all the [^14^C]pectin was wall-bound), when the intensity of the monomeric [^14^C]RG-II spot remained almost constant, the dimeric [^14^C]RG-II increased slightly, especially in *Rosa* (Figs 5B and 6). This indicates that, whereas the majority of boron bridging was occurring intraprotoplasmically, some additional dimerization was gradually continuing post-secretion, in the apoplast.

## DISCUSSION

### Satisfactory systems for pulse labelling RG-II with ^14^C

Heterotrophic cell suspensions of *Rosa* and arabidopsis cultured with glycerol as their sole carbon source were excellent model systems for pulse-radiolabelling of RG-II and the other pectic domains. Optimal polysaccharide labelling was obtained with ~5- and 1-d-old cultures of *Rosa* and arabidopsis respectively. These cells grow well in glycerol-based media; nevertheless, their preferred substrates are hexoses or sucrose (Sharples and Fry, 2007). Therefore, when traces of [^14^C]glucose are supplied to glycerol-grown cultures, the cells avidly take up the radiolabelled substrate and metabolize it to, among other products, wall polysaccharides. The cultures had consumed almost all the exogenous [^14^C]glucose after ~90 min; thereafter, relatively little further radiolabelling of polysaccharides occurred. Thus, it was possible to trace the ‘careers’ of a cohort of newly synthesized polysaccharide molecules in living cells and observe the kinetics of RG-II dimerization.

Throughout our work in this experimental system, there was a surprisingly high proportion of monomeric RG-II, especially as seen in the radiolabelled bands, with the [^14^C]monomer exceeding [^14^C]dimer (Figs 2, 5B and 6). Some previous studies in various other systems have often shown in the order of 90 % dimer. The difference between the current data and some prior observations is currently inexplicable. However, it cannot be due to preferential loss of radiolabelled dimers during fixing and washing of the gels after electrophoresis; by contrast, the monomer, being smaller and more diffusible, would be more prone to leaching out of the gel than the dimer, thus leading to an overestimation of the [^14^C]dimer.

### Most boron bridging of [^14^C]RG-II occurs intraprotoplasmically

As expected in these relatively short-term experiments, the total RG-II concentration (radioactive plus non-radioactive) in the *Rosa* and arabidopsis cells showed no consistent time-dependent changes (Fig. 6, silver-stained gels). Both monomeric and boron-bridged dimeric RG-II were detectable, even in the *Rosa* culture, whose medium contained only 3.3 µm boric acid.

Both monomeric and dimeric RG-II domains were being detectably radiolabelled within 4 min of [^14^C]glucose feeding (Fig. 5B), emphasizing the rapidity of this metabolic pathway (as noted with [^14^C]fructose feeding; Sharples and Fry, 2007). Thus, the pathway from exogenous [^14^C]glucose, via intracellular [^14^C]sugar phosphates and [^14^C]sugar nucleotides, to [^14^C]RG-II required <4 min (Figs 5B and 6). The extent of ^14^C-labelling of total RG-II (monomer + dimer) increased between 4 and 120 min at an approximately linear rate (Fig. 5B), representing *de novo* [^14^C]RG-II biosynthesis.

The key observation is that between 4 and 120 min the radiolabelling of boron-bridged RG-II dimers paralleled that of the monomers (Figs 5B and 6). The [^14^C]dimer is faintly visible at all time points, including 4 min, in both plant species (Figs 6 and Supplementary Data, Fig. S4); the corresponding quantitative data in Fig. 5B at 4 min may demonstrate this more convincingly. There was a negligible lag between the synthesis of an RG-II domain and its cross-linking via a boron bridge. This 4-min time span should be compared with the transit time of 15–20 min for polysaccharides, newly synthesized in the Golgi bodies, to reach the cell wall and/or extracellular medium in similar cells (Northcote and Pickett-Heaps, 1966; Dauwalder and Whaley, 1974; Gardiner and Chrispeels, 1975; Robinson *et al.*, 1976; Fry, 1987, 2004; Myton and Fry, 1994; Fry *et al.*, 2000). Thus, the dimeric [^14^C]RG-II domains observed at 4, 8 and 16 min (Figs 5B and 6) were still largely intraprotoplasmic. [Although total (radioactive + non-radioactive) RG-II in the cell wall greatly exceeded that in the Golgi system, essentially all the radioactive RG-II molecules would have still been intraprotoplasmic up to 16 min after ‘time 0’.] Extensive boron bridging of newly synthesized RG-II was evidently occurring in the Golgi system.

Looking at Figs 3C and 5B, and comparing the [^14^C]polysaccharides present at 16 min (vast majority intraprotoplasmic) vs. 1020 min (vast majority wall-located), we conclude that ~11–22 % of the eventual total [^14^C]polysaccharide was intraprotoplasmic at 16 min.

We conclude that the boron bridging of RG-II was occurring predominantly within the protoplast, within 4 min of polysaccharide domain synthesis. These kinetics refine the conclusion of Chormova *et al.* (2014), who had shown that boron bridging occurred a short time after polysaccharide synthesis but were unable to say whether this was intraprotoplasmically or at the moment of entry into the apoplast, or both.

### A minority of boron bridging of [^14^C]RG-II occurs post-secretion

Between 120 and 1020 min, during which period there was little remaining extracellular [^14^C]glucose, negligible intracellular [^14^C]glucose 6-phosphate, and little further [^14^C]homogalacturonan and [^14^C]RG-I synthesis, the average rate of total [^14^C]RG-II accumulation was reduced to about 8 and 6 % of the 0–120-min period in *Rosa* and arabidopsis respectively (see Table inset in Fig. 5B). Therefore, the 120–1020-min interval allowed us to observe very predominantly just the ‘ageing’ of [^14^C]RG-II domains pre-formed before 120 min.

During this 120–1020-min period (i.e. ~8–70 times the secretory transit time), the intensity of the monomeric [^14^C]RG-II spot remained relatively constant, but dimeric [^14^C]RG-II increased slightly, especially in *Rosa* (Figs 5B and 6). This indicates that although the majority of boron bridging was occurring intraprotoplasmically, some additional dimerization was gradually continuing post-secretion, i.e. within the apoplast. Note that the Golgi-localized dimerization is what can be seen in the first 16 min, whereas the ultimate dimer : monomer ratio takes 1020 min to be achieved – hence our assertion that dimerization occurs ‘at a reduced rate after secretion’. These kinetics again refine the conclusion of Chormova *et al.* (2014), in that we now report *low*, rather than *zero*, boron bridging after RG-II secretion into the apoplast of *Rosa*.

Contrasting with both the present work and the data of Chormova *et al.* (2014), it had earlier been reported that, when boric acid was suddenly re-supplied to boron-starved *Chenopodium* cell cultures (Fleischer *et al.*, 1999) or *Cucurbita* leaves (Ishii *et al.*, 2001), *most* of the ‘pre-existing’ wall-bound RG-II domains became boron-bridged. In the *Cucurbita* leaves, it may be questioned whether the RG-II which became dimerized was all pre-existing and wall-bound because significant dimerization was not observed until 5 h after the addition of 25 µm boric acid (Ishii *et al.*, 2001), a time span which was probably sufficient for some *de novo* RG-II biosynthesis, resulting in new RG-II molecules which could have become boron-bridged intraprotoplasmically.

In the boron-starved *Chenopodium* cell cultures, the dimerization of monomeric RG-II domains was observed within 10 min of the addition of 100 µm boric acid. Also, changes in wall porosity (attributed to RG-II cross-linking) occurred within 10 min of the addition of 10 µm boric acid, even under anaerobic conditions and thus presumably concerning pre-existing wall-bound RG-II (Fleischer *et al.*, 1999). The lack of interference by anaerobiosis is relevant because Chormova *et al.* (2014) found that only newly synthesized RG-II became boron-bridged – a process blocked by anaerobiosis and respiratory inhibitors which prevent *de novo* polysaccharide synthesis.

To explain the discrepancy between cell cultures of *Chenopodium* (Fleischer *et al.*, 1999) and *Rosa* and arabidopsis (Chormova *et al.*, 2014, and present work), it may be relevant that the RG-II domains of *Chenopodium* were extractable from the cells in cold phosphate buffer (prior to EPG digestion), and were therefore not firmly integrated into the wall architecture. The *Rosa* and arabidopsis pectin were not buffer-extractable. It might be suggested that the loosely bound RG-II domains in *Chenopodium* were manoeuvrable enough to dimerize in the apoplast whereas the firmly wall-integrated domains in *Rosa* and arabidopsis were not. Nevertheless, in the work of Chormova *et al.* (2014), exogenous *soluble* RG-II molecules, which are clearly manoeuvrable, were not capable of becoming boron-bridged when added to the medium of cultured *Rosa* cells in the presence of 3.3 µm boric acid.

It remains unclear why boron bridging occurred in the *Chenopodium* apoplast (and probably the *Cucurbita* leaf apoplast) but not in the *Rosa* apoplast (Chormova *et al.*, 2014) or only slightly in the *Rosa* and arabidopsis apoplast (present work). One possibility might be the different boric acid concentrations used in different experiments: 10–100 µm in *Chenopodium* and 25 µm in *Cucurbita* (but supplied via the roots and possibly becoming more concentrated in the leaves owing to the transpiration stream), vs. the much lower concentration of 3.3 µm in *Rosa* cultures. It is possible that higher concentrations facilitate post-secretion dimerization. Nevertheless, in the present work, boric acid was supplied at 3.3 and 100 µm for the *Rosa* and arabidopsis cultures respectively, but both cultures gave rather similar data evidencing only slight post-secretion dimerization.

### Remaining unknowns and implications

The boron bridging of pectic RG-II domains in *Rosa* and arabidopsis cell cultures has been shown to occur predominantly within the Golgi system, prior to release into the apoplast, and to continue at a much reduced rate after secretion.

It remains to be determined whether the RG-II domains that participate in boron bridging were already part of the covalent structure of complex pectin molecules, or were free RG-II molecules within the Golgi system. This information is lost when the samples are digested with EPG. Indeed, it is not yet known whether RG-II is (1) synthesized in free form and then has its GalA_*n*_ backbone covalently bonded to a homogalacturonan or RG-I domain to form a continuous backbone, or (2) assembled by glycosyltransferases onto an existing or nascent homogalacturonan backbone.

Since RG-II domains are now seen to become dimerized within the Golgi system, we conclude that the compound which donates the boron atom for bridge formation is located within the Golgi lumen. This boron ‘donor substrate’ may simply be boric acid, taken up via the BOR1 transporter (Takano *et al.*, 2002; Noguchi *et al.*, 2003) or it may be a complex with a cationic chaperone such as a histidine-rich arabinogalactan-protein (Chormova and Fry, 2016; Sanhueza *et al.*, 2022) or with a glycolipid (Voxeur and Fry, 2014). There are many remaining unknowns concerning the boron bridging of RG-II, but the present work has established that the whole machinery for this process must be present within the Golgi system.

We also cannot yet answer the question of whether boron-bridged RG-II domains promote wall plasticity and thus cell expansion, or contribute to wall architecture and perhaps thus restrain cell expansion. However, knowing where in the cell (and the related issue of when in the ‘career’ of an RG-II domain) the bridging occurs will help us to visualize the possible biological roles of boron bridging.

## CONCLUSIONS

The literature contains conflicting hypotheses concerning the kinetics and subcellular localization of boron bridging of the RG-II domains of pectin. It is therefore difficult to reach concrete conclusions on the precise mechanism and roles of boron bridging. It is agreed, however, that such bridging is essential for normal plant growth, especially cell expansion. RG-II is synthesized in the Golgi apparatus, but boron bridging could occur at any or all of: (1) within the Golgi system during or immediately after biosynthesis, (2) concurrently with secretion through the plasma membrane, and (3) within the cell wall potentially a long time post-secretion. We therefore explored the kinetics of RG-II bridging.

In the cell cultures of *Rosa* and arabidopsis, a high proportion of the newly synthesized (pulse-radiolabelled) RG-II domains became boron-bridged almost immediately – within well under 4 min, compared with the ~15 min required for secretion into the apoplast. Little additional boron bridging occurred in the following ~90 min, i.e. within an hour of secretion. However, some limited further boron bridging did occur in the following 15 h, i.e. while the cohort of [^14^C]RG-II domains being traced were located in the cell wall. We conclude that the kinetics of boron bridging of RG-II was biphasic – first (mostly) Golgi-localized, and second (less extensively) in the apoplast (cell wall). In other systems, the latter phase was reported (Fleischer *et al.*, 1999; Ishii *et al.*, 2001) to be more prevalent than in the two cell cultures studied by us.

RG-II does not readily bind boron when simply incubated in a boric acid solution at physiological pH; it is clear that certain cellular agents assist in the RG-II/boron interaction, proposed to be histidine-rich glycoproteins (as cationic chaperones; Chormova and Fry, 2016) and glycolipids (e.g. glycosylinositol phosphorylceramides; Voxeur and Fry, 2014). It would be interesting to discover precisely what these agents are, and which of them are located where in the cell. The findings reported here show where in the cell (and thus when in the ‘career’ of an RG-II domain) boron bridging occurs, helping to define the possible biological roles of RG-II dimerization and the probable localization of boron-donating glycoproteins and glycolipids.

## SUPPLEMENTARY DATA

Supplementary data are available online at https://academic.oup.com/aob and consist of the following.

**Fig. S1.** ^14^C-Labelling of RG-II by differently aged cultures.

**Fig. S2.** [^14^C]Glucose is removed from the culture medium accompanied by release of traces of extracellular radioactive metabolites.

**Fig. S3.** Characterization of the intermediary product, radiolabelled during exogenous feeding of [^14^C]Glc and released from AIR by EPG.

**Fig. S4.** Time-course of ^14^C-labelling of monomeric and dimeric RG-II domains: replicate.

mcac119_suppl_Supplementary_Figures
