# Supplementary material for: Boron bridging of rhamnogalacturonan-II in Rosa and arabidopsis cell cultures occurs mainly in the endo-membrane system and continues at a reduced rate after secretion
Source: Ann Bot. 2022 Sep 16;130(5):703–15. doi: 10.1093/aob/mcac119 (PMC9670748; doi:10.1093/aob/mcac119)
Supplement: mcac119_suppl_Supplementary_Figures [file mcac119_suppl_supplementary_figures.pptx]

## Slide 1
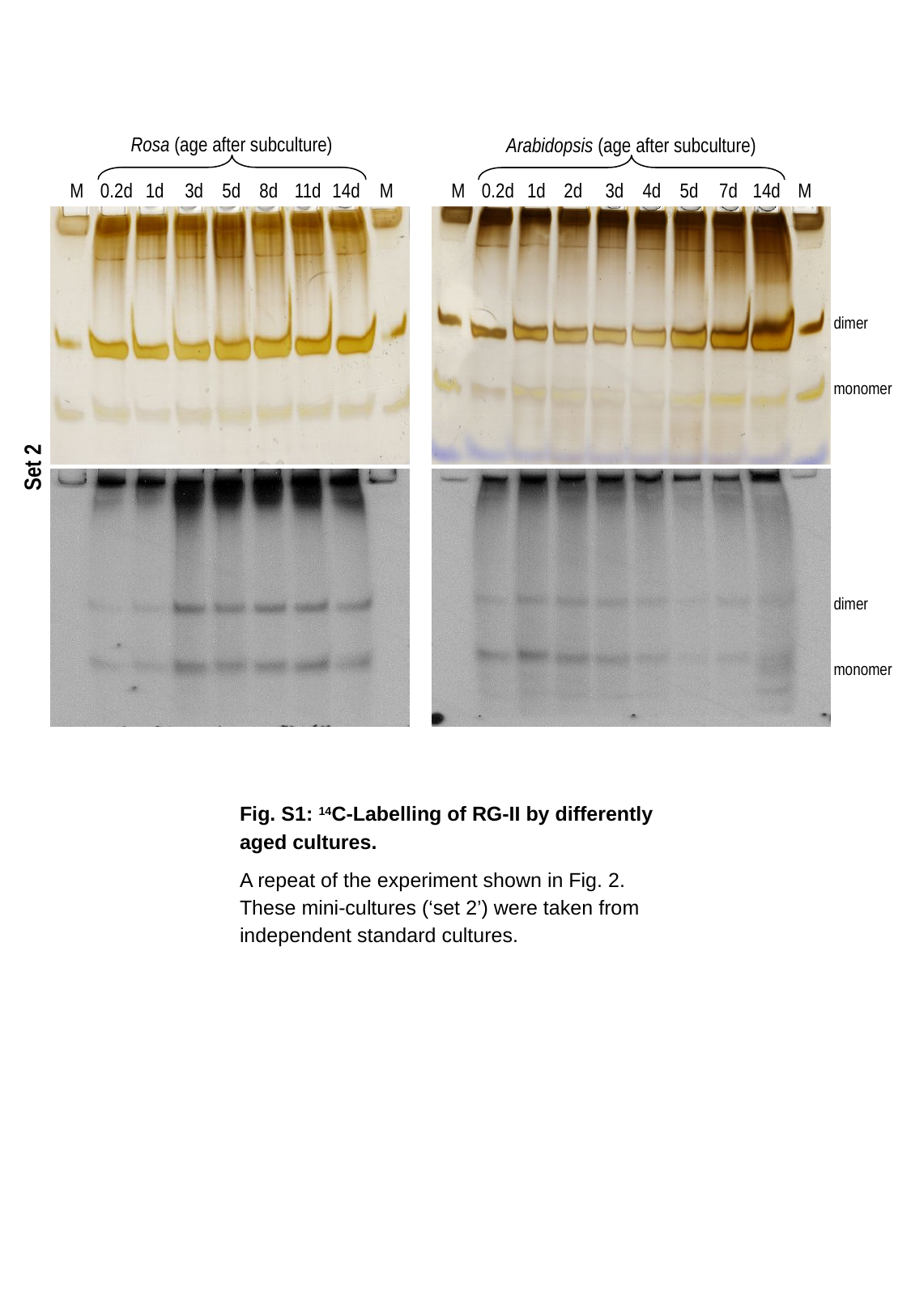

Rosa (age after subculture)
Arabidopsis (age after subculture)
M
0.2d
1d
3d
5d
8d
11d
14d
M
M
0.2d
1d
2d
3d
4d
5d
7d
14d
M
dimer
monomer
Set 2
dimer
monomer
Fig. S1: 14C-Labelling of RG-II by differently aged cultures.
A repeat of the experiment shown in Fig. 2. These mini-cultures (‘set 2’) were taken from independent standard cultures.

## Slide 2
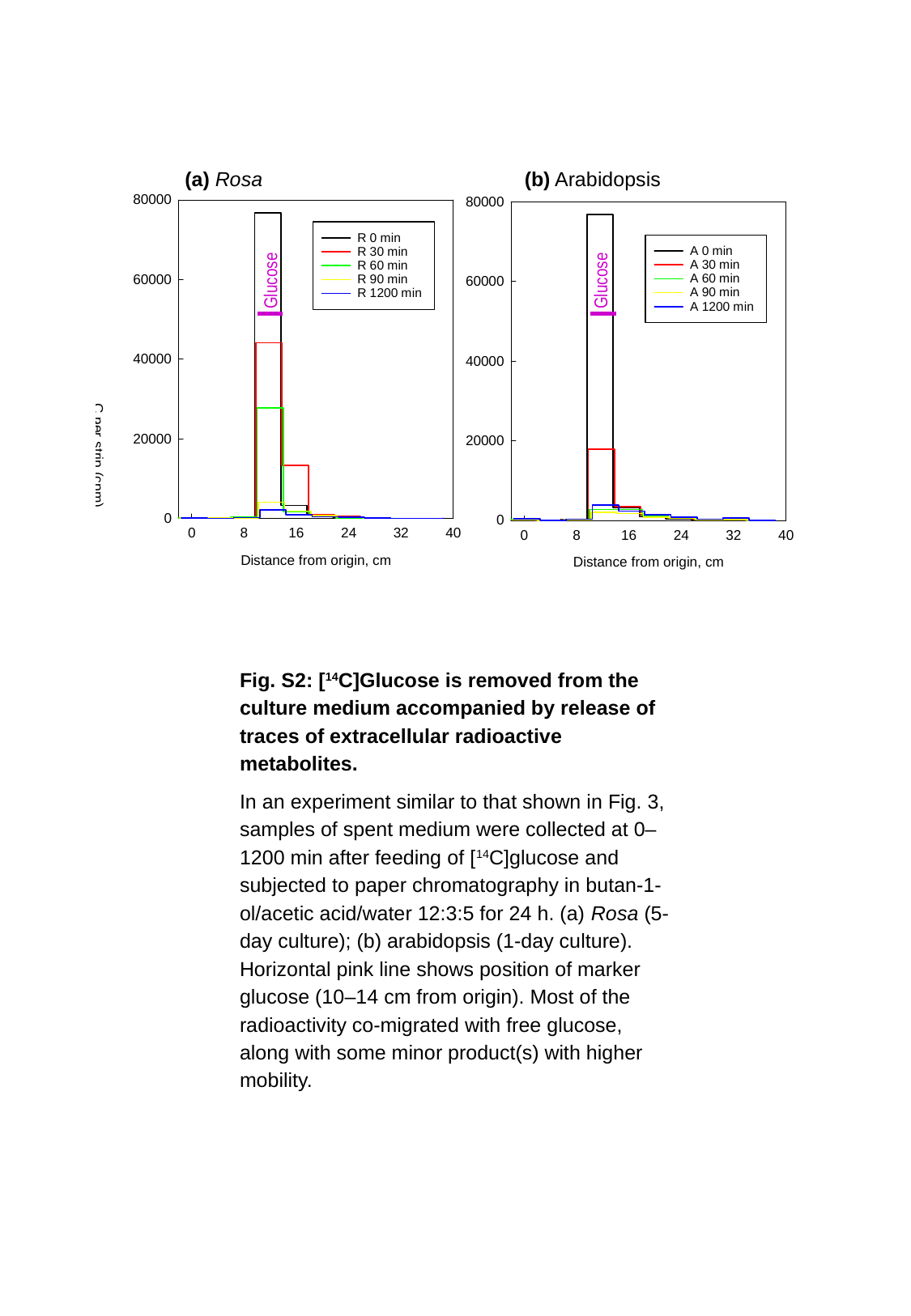

(a) Rosa
(b) Arabidopsis
Glucose
Glucose
Fig. S2: [14C]Glucose is removed from the culture medium accompanied by release of traces of extracellular radioactive metabolites.
In an experiment similar to that shown in Fig. 3, samples of spent medium were collected at 0–1200 min after feeding of [14C]glucose and subjected to paper chromatography in butan-1-ol/acetic acid/water 12:3:5 for 24 h. (a) Rosa (5-day culture); (b) arabidopsis (1-day culture). Horizontal pink line shows position of marker glucose (10–14 cm from origin). Most of the radioactivity co-migrated with free glucose, along with some minor product(s) with higher mobility.

## Slide 3
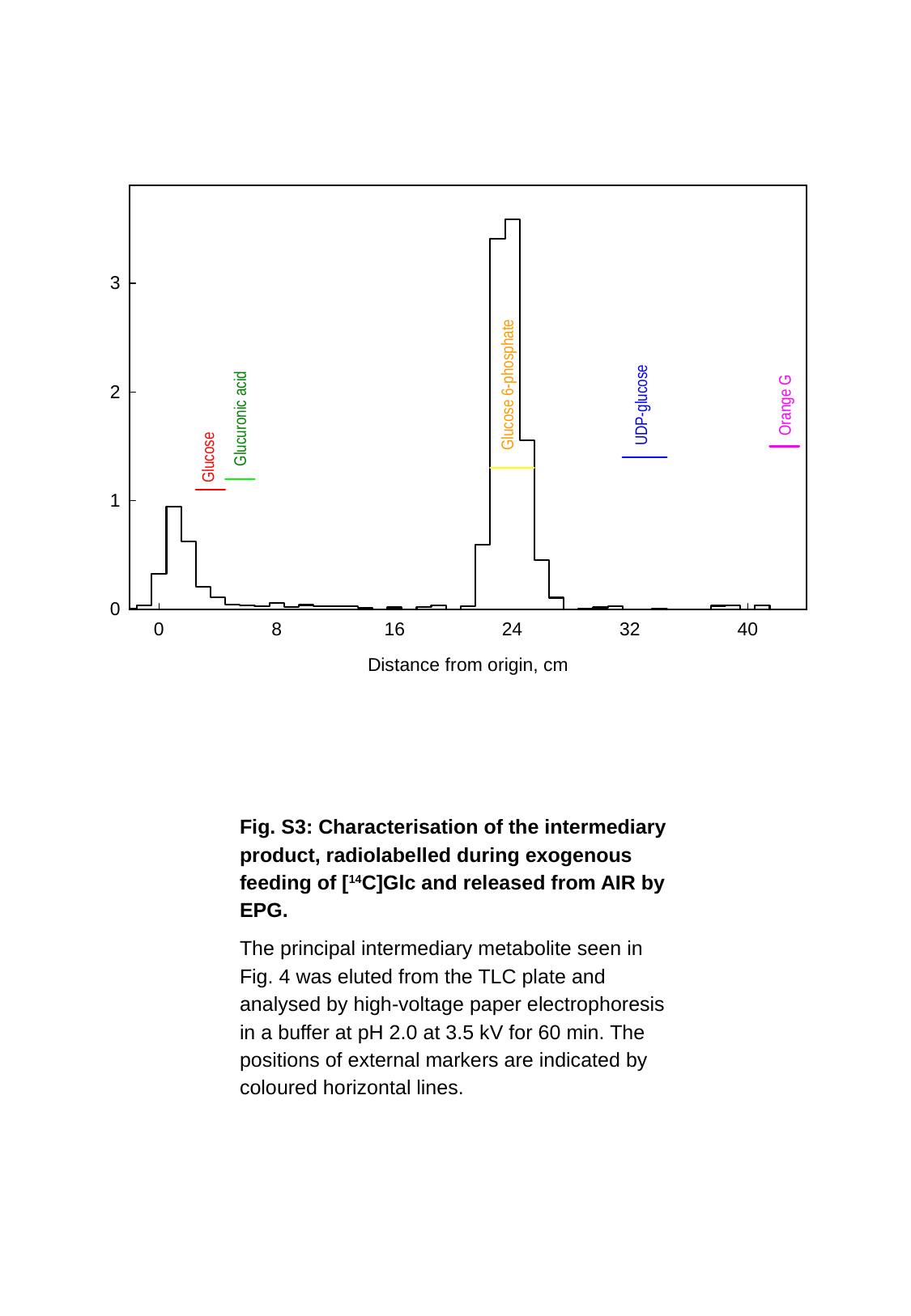

Glucose 6-phosphate
UDP-glucose
Orange G
Glucuronic acid
Glucose
Fig. S3: Characterisation of the intermediary product, radiolabelled during exogenous feeding of [14C]Glc and released from AIR by EPG.
The principal intermediary metabolite seen in Fig. 4 was eluted from the TLC plate and analysed by high-voltage paper electrophoresis in a buffer at pH 2.0 at 3.5 kV for 60 min. The positions of external markers are indicated by coloured horizontal lines.

## Slide 4
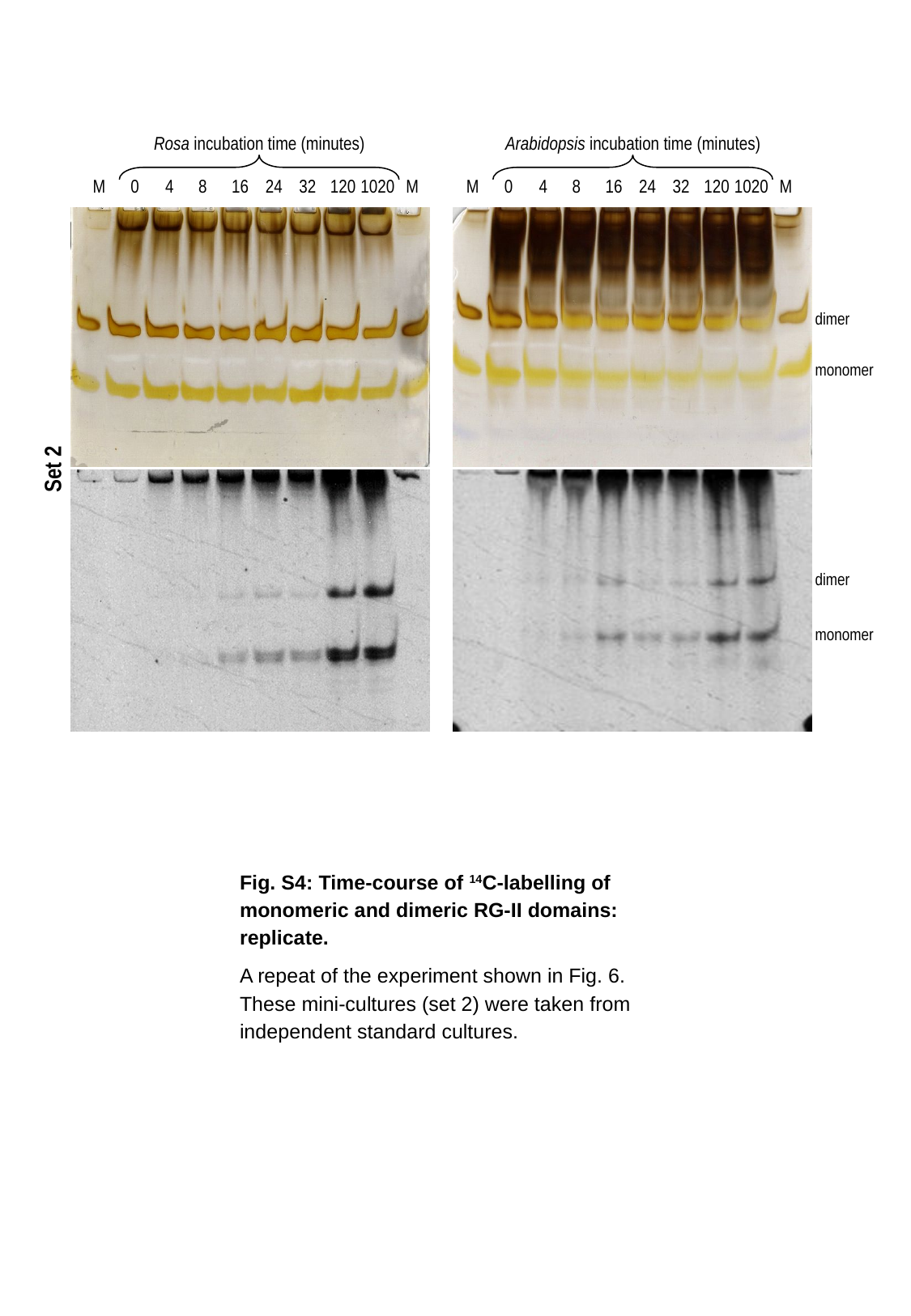

Rosa incubation time (minutes)
M
0
4
8
16
24
32
120
1020
M
Arabidopsis incubation time (minutes)
M
0
4
8
16
24
32
120
1020
M
dimer
monomer
Set 2
dimer
monomer
Fig. S4: Time-course of 14C-labelling of monomeric and dimeric RG-II domains: replicate.
A repeat of the experiment shown in Fig. 6. These mini-cultures (set 2) were taken from independent standard cultures.
